# Juvenile Resilience and Adult Longevity Explain Residual Populations of the Andean Wax Palm *Ceroxylon quindiuense* after Deforestation

**DOI:** 10.1371/journal.pone.0074139

**Published:** 2013-10-23

**Authors:** María José Sanín, Fabien Anthelme, Jean-Christophe Pintaud, Gloria Galeano, Rodrigo Bernal

**Affiliations:** 1 Instituto de Ciencias Naturales, Universidad Nacional de Colombia, Bogotá, Distrito Capital, Colombia; 2 Botanique et Bioinformatique de l’Architecture des Plantes, Recherche Agronomique pour Le Développement, Institut de recherche pour le développement, Montpellier, Hérault, France; 3 Botanique et Bioinformatique de l’Architecture des Plantes and Diversité Adaptation et Développement des plantes, Institut de recherche pour le développement, Montpellier, Hérault, France; Centro de Investigación y de Estudios Avanzados, Mexico

## Abstract

Wax palms are an important element of the cloud forests in the tropical Andes. Despite heavy deforestation, the density of adults seems to be similar in deforested pastures as in forests. We aimed to infer the mechanisms responsible for this apparent resilience in pastures and we tested two hypotheses to explain it: 1) adult palms survived in pastures because they were spared from logging, and 2) adults occurred in pastures through the resilience of large juvenile rosettes, which survived through subterranean meristems and later developed into adults. For this purpose, we characterized the demographic structure of *C. quindiuense* in a total of 122 plots of 400 m^2^ in forests and pastures at two sites with contrasted land use histories in Colombia and Peru. Additionally, we implemented growth models that allowed us to estimate the age of individuals at four sites. These data were combined with information collected from local land managers in order to complete our knowledge on the land use history at each site. At two sites, the presence of old individuals up to 169 years and a wide age range evidenced that, at least, a portion of current adults in pastures were spared from logging at the time of deforestation. However, at the two other sites, the absence of older adults in pastures and the narrow age range of the populations indicated that individuals came exclusively from rosette resilience. These interpretations were consistent with the land use history of sites. In consequence, the combination of the two hypotheses (spared individuals and rosette resilience) explained patterns of *C. quindiuense* in pastures on a regional scale. Regeneration through subterranean meristems in palms is an important, yet overlooked mechanism of resilience, which occurs in a number of palm species and deserves being integrated in the conceptual framework of disturbance ecology.

## Introduction

Deforestation in the tropics has accelerated in recent years, and its progress has gained worldwide attention [1,2]. Along with climate change, it is the central cause of current and predicted loss of biodiversity and of the ecosystem services it sustains [3,4,5]. Accordingly, the level of resilience expressed by tropical forest species in deforested, burned, or grazed areas is a crucial driver of biodiversity maintenance and understanding the patterns and mechanisms of resilience is a critical scientific challenge [6,7]. The best re-colonizing plant species after deforestation and related damages are ruderal strategists sensu Grime [8], which use specific mechanisms to tolerate strong disturbances, such as resprouting [9], resistance to fire (e.g., *Acrocomia aculeata* [10,11],; *Attalea* spp. [12]), or physical/chemical defenses against herbivory [13]. However, these species represent a very low portion of the overall forest plant diversity, and they rarely include trees. Moreover, despite producing shade with their foliage, which is expected to have positive effects on the establishment of other forest species [14,15], they often tend to form dense, monopolistic populations that inhibit the development of other species [16,17]. For example, in the Andean cloud forest, recolonization of gaps and cleared areas is commonly achieved by *Chusquea* spp. (scandent bamboos), which develop long-lived dense thickets impeding secondary succession, until the occurrence of die-off following synchronized flowering events [18,19]. Consequently, the survival of forest plant communities confronted to the multiple effects of deforestation mostly depends on the resilience capacities of arborescent species themselves. The extent to which these species are resilient becomes even more important in the case of "keystone species", “foundation species” or “umbrella species”, whose presence sustains the establishment of a large array of other taxa, including animals and plants [20,21].

In tropical forests, an interesting but understudied pattern of tree resilience has been observed in several species of the palm genus *Ceroxylon* (Arecaceae), an iconic, “umbrella” taxon of the neotropical Andean forests sustaining many ecosystem services and having important positive effects on various components of the biodiversity [10,22]. Some of the largest *Ceroxylon* populations are located in the vicinity of human settlements and thus confront the deleterious effects of deforestation, logging, burning, and large herbivores. Nevertheless, the adult density of *Ceroxylon* in deforested pastures seems to approach that of stands within adjacent forests, suggesting that the palms might be, to some extent, resilient to the effects of deforestation. However, *Ceroxylon* spp. are dioecious, long-lived palms, which develop a medium-sized to tall solitary stem without resprouting capacities, and with seedlings that do not tolerate the exposed conditions of open areas [22]. Accordingly, this taxon does not follow the characteristics of a ruderal species, and the relative high frequency of adults in deforested pastures is rather unexpected. 

We aimed at exploring the mechanisms by which *Ceroxylon* persists under the harsh environmental conditions of deforested mountain areas. For this purpose we investigated two hypotheses. First, we tested to what extent adult palms survived in pastures because they are spared from logging, having established in the formerly existing forest. Such hypothesis has been proposed recently in the case of the species *C. echinulatum* in Ecuador [22] and has been reinforced by interviews of local inhabitants explaining that *Ceroxylon* was regularly spared in pastures for various reasons, including keeping it as a construction material to be eventually used or the difficulty to cut the stems (Fabien Anthelme, unpublished data). Second, we explored the hypothesis that *C. quindiuense* may maintain adult populations in deforested areas through the resilience of large juvenile rosettes. This hypothesis is sustained by the fact that, once they reach the stage of large rosettes, individuals of *Ceroxylon* develop a subterranean meristem (underground stem), which is protected by the thick and tightly arranged enclosing leaf sheaths [23,24].

To achieve this goal, we studied populations of *C. quindiuense* in forests and pastures at two sites with contrasting land use scenarios in Peru (recent deforestation) and Colombia (old deforestation). We examined the population structure and coupled demographic data with an estimation of the age of individuals, seeking evidence of a relationship between the history of deforestation and the age of populations. In light of the results found, we explored predictions on the long-term conservation of *Ceroxylon*, by comparing a series of deforestation scenarios.

## Methods

### Target taxon

The genus *Ceroxylon* (wax palms) makes up a group of twelve species endemic to the tropical Andes (from Venezuela to Bolivia), where they are adapted to the relatively cold temperatures of the mountain cloud forests [25]. Most of *Ceroxylon* species are gregarious and reach high population densities, constituting a dominant structural element of the highly diverse cloud forest. For these reasons, and because they produce a high amount of fleshy fruits attractive for a large array of birds, rodents, and other mammals (e.g. *Dasypus novemcinctus*, *Sciurus granatensis*, *Odocoileus virginianus*, *Pecari tajacu*, *Eira barbara*) *Ceroxylon* is considered a keystone taxon with outstanding positive impact on the functioning of the cloud forest [22,25]. 

**Figure 1:**
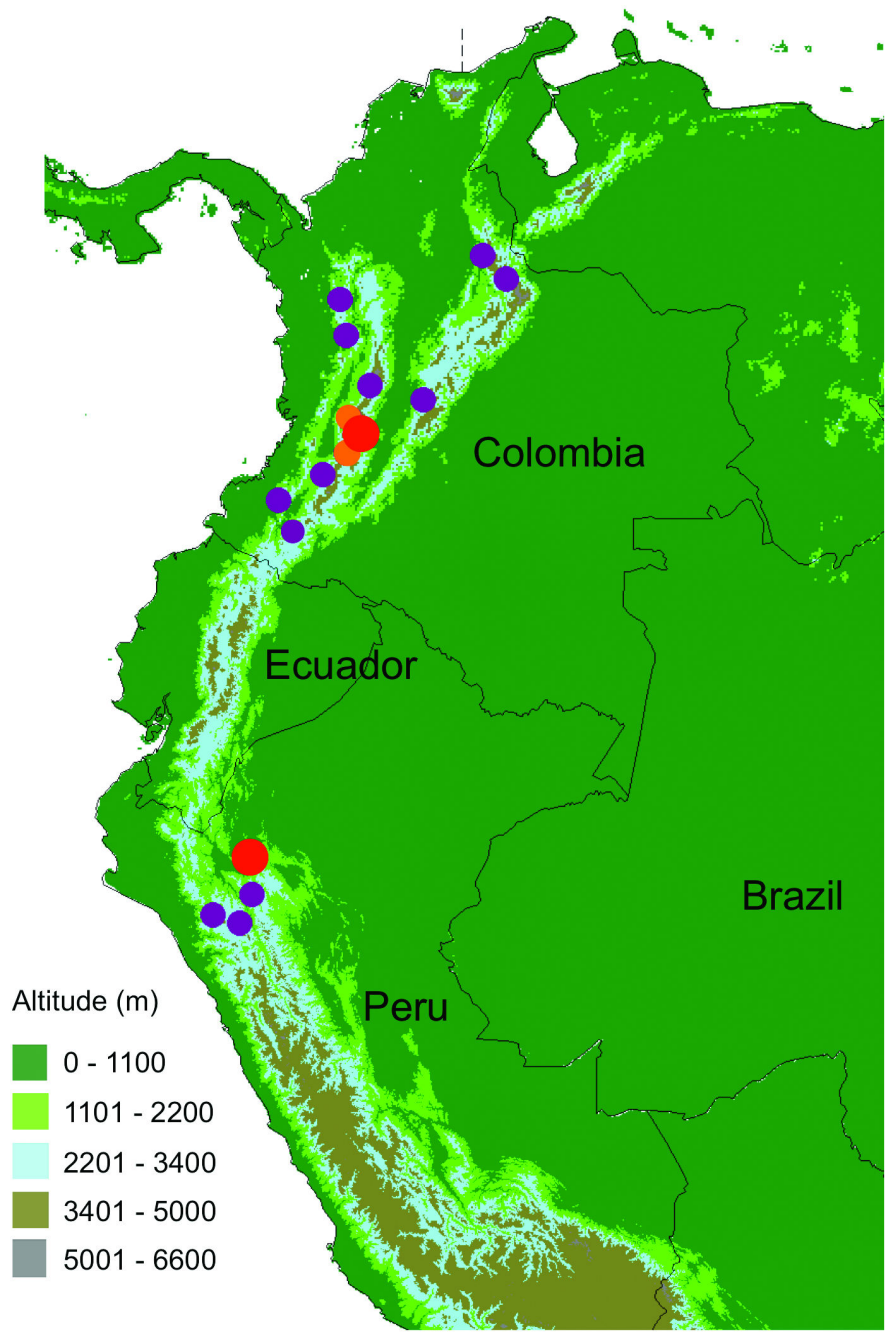
Distribution of *Ceroxylon quindiuense* [25]. Small purple dots indicate presence of populations; large red dots indicate sites where plots were established and growth models applied; medium-sized orange dots indicate the two additional sites where growth models were applied.

*C. quindiuense* is a tall, single-stemmed palm that can reach 52 m in height [25]. It is the tallest palm in the world and Colombia’s National Tree [10]. It forms dense stands in the cloud forests of the three cordilleras of Colombia and disconnectedly in northern Peru between (1600-) 2000—2700 (-3200) m (Figure 1) [26]. It is considered endangered (EN) in Colombia according to IUCN criteria [27], due to the reduction of its habitat and to the low regeneration rates in grazed areas. For example, since the second half of the 19^th^ century, the areas of cloud mountain forest in Colombia are submitted to a typical slash-and-burn regime, followed by cultivation of grasses for cattle (dairy) grazing or crops such as corn, potatoes, tomatos, peas, and beans. The land is periodically burnt every 5—10 years to restart a cultivation cycle [28]. 

### Study sites

**Figure 2:**
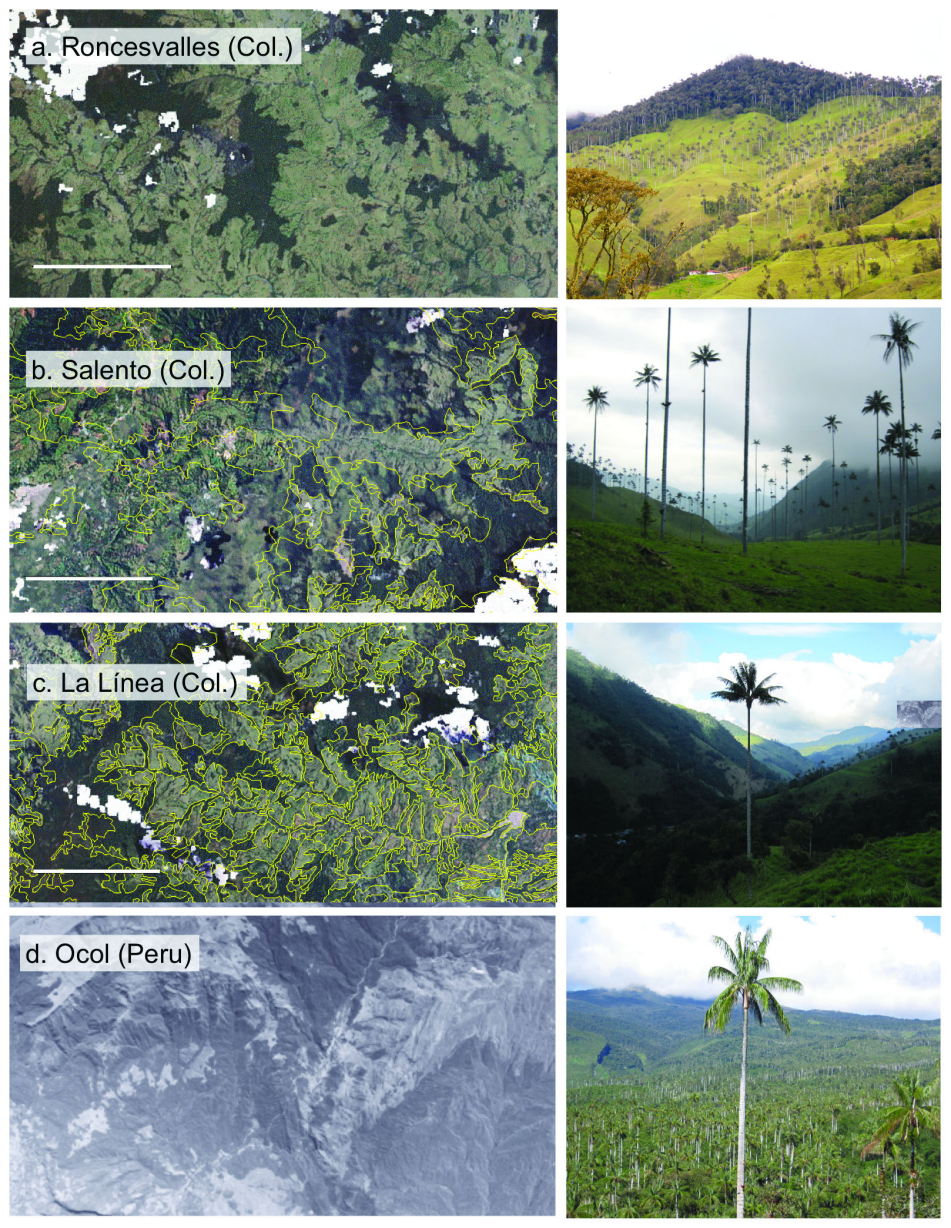
Satellite images and general sceneries revealing patterns of deforestation at each studied site. Left, images of the Instituto Agustín Codazzi Geoportal, scale bars = 4.67 km (a-c), and institutional aerial photograph without scale of the Instituto de Investigación de la Amazonía Peruana, standing in representation of general landscape composition since satellite images of this site were not available for publication (d). Right, general sceneries of the four studied sites (including palms). In Roncesvalles, a general matrix of pastures surrounds several fragments of forest; at Ocol, a general fragment of forest surrounds several fragments of pastures; at Salento and La Línea, deforestation extent resembles that of Roncesvalles, but there are more fragments of forest, and these are more connected; percent of forest cover at each site is indicated in parenthesis: Roncesvalles (11%), Ocol (84%), Salento (46%) La Línea (42%).

We studied the ecological structure of populations of *C. quindiuense* at two distant sites with contrasting forest cover, in Colombia and in Peru (Figure 1: large dots; Figure 2a, 2d). In Colombia, we established plots at Roncesvalles, Department of Tolima, in the Central Cordillera (04°02’ N, 75°36’ W), between 2500 and 3000 m a.s.l. Landscape at this site consists of small to medium-sized forest fragments embedded in a dominant matrix of pastures (Figure 2a). The forest remnants have a canopy layer up to 20 m high, with some emergent trees up to 30 m high and 35-40 cm dbh, typical of mature forests.These include *Quercus humboldtii* (Fagaceae), *Cedrela montana* (Meliaceae)*, Weinmania rollottii* (Cunnoniaceae), *Hyeronima* sp. (Euphorbiaceae), *Billia rosea* (Sapindaceae), *Drymis granadensis* (Winteraceae), and several species of Lauraceae and Meliaceae. Nevertheless, most of the woody components are typical of secondary forest, such as *Cytharexylum subflavescens* (Verbenaceae)*, Palicourea angustifolia* (Rubiaceae), *Myrsine coriacea* (Primulaceae)*, Clethra* sp. (Clethraceae), *Lozania mutisiana* (Lacistemataceae)*, Clusia* sp. (Clusiaceae), *Miconia lehmannii* and *Miconia psychrophila* (Melastomataceae), and the arborescent fern *Dicksonia sellowiana*, among others. The understory layer is composed mainly by herbs as *Lycianthes radiata* (Solanaceae), *Habracanthus* sp. (Acanthaceae), *Anthurium* sp. (Araceae), *Pilea* spp. and *Peperomia* spp. (Piperaceae), *Baccharis* sp. and several species of Asteraceae, seedlings and sapling of shrubs and canopy trees, and in some areas, *Chusquea* sp. (Poaceae) [29,30]. Extensive deforestation in this area occurred at in the late 19^th^ and early 20^th^ centuries, during the process known as *Antioqueño Colonization* [31,32]. 

In Peru, plots were established in Ocol, Department of Amazonas (06°15’46” S 77°34’06” W), between 2200 m and 2800 m. Landscape at this site consists of small areas of pasture in a dominant matrix of forest (Figure 2d). The forest is composed by trees reaching up to 20-30 m high and to 40 cm dbh, some of them typical of mature forests like *Nectandra* sp., *Ocotea* sp. and *Persea* sp. (Lauraceae), *Licania* sp. (Chrysobalanaceae), *Weinmannia* sp. (Cunnoniaceae), and many trees and shrubs of secondary forest like *Alnus acuminata* (Betulaceae), *Clusia* sp. (Clusiaceae), *Pourouma* sp. and *Cecropia* sp. (Urticaceae), *Calyptranthes* sp. (Myrtaceae), and *Ficus* sp. (Moraceae), as well as many shrubs and some arborescent ferns (*Alsophylla* sp., *Cyathea* sp). The forest understory is composed by many herbs of *Anthurium* spp. (Araceae), *Piper* spp. and *Peperomia* spp. (Piperaceae), Solanaceae, *Baccharis* sp., and other Asteraceae, seedlings and saplings of woody species, and some woody scandent shrubs of *Rubus* sp. (Rosaceae) and *Chusquea* sp. (Poaceae) [33]. According to Mateo & Cornejo [33], pastures are restricted to the lower areas of the valley and were cleared starting in the 1970’s, whereas upper, forested slopes in Ocol were exploited mainly through selective logging. Despite their different land use histories, both sites have dense patches of *C. quindiuense*, and were therefore considered ideal settings to compare. The abiotic and biotic factors in each plot are included in Table 1, and the full data for each plot are comprised in the Supporting Information (Datasets S1-S2). 

**Table 1:** Biotic and abiotic structure of *Ceroxylon quindiuense* plots, among sites and habitats in Colombia and Peru.

*Site*	Roncesvalles, Colombia		Ocol (Peru)	
*Habitat*	Forest	Pasture	Forest	Pasture
*Ceroxylon* Diameter (cm) (***/***)	37.23 ± 0.47^b^	44.26 ± 0.96**^a^**	34.95 ± 0.38**^c^**	37.75 ± 0.81**^b^**
Basal area *Ceroxylon* (m^2^/ha) (-/***)	78.56 ± 21.71**^b^**	27.03 ± 9.08**^a^**	96.07 ± 10.89**^b^**	19.41 ± 4.62**^a^**
Basal area trees (m^2^/ha) (***/***)	837.78 ± 78.50^b^	1.64 ± 0.91^d^	1425.92 ± 77.20^a^	29.63 ± 6.14^c^
% of basal area by *Ceroxylon* (***/***)	11.1 ± 3.0**^a^**	90.2 ± 16.8**^b^**	6.5 ± 0.73**^a^**	40.1 ± 2.3**^b^**
Altitude (m) (***/***)	2777.8 ± 16.7**^a^**	2705.6 ± 13.9**^b^**	2422.9 ± 16.2**^c^**	2310.3 ± 21.7**^d^**
Slope (°) (***/*)	23.58 ± 1.30**^a^**	19.63 ± 1.55**^ab^**	18.45 ± 1.25**^b^**	13.35 ± 1.52**^c^**

Differences among treatments are analyzed with fixed effects models (habitat nested within site; into brackets: significance of site/ habitat), standard errors after each value. Common letters indicate no difference among pairs of treatments within each site (p < 0.05, two-sample t-tests, hypothesis: not equal, adjusted for ties).

In order to calculate the age of adult palms, we collected data at the two sites described above plus two additional sites in Colombia displaying the same pattern of *C. quindiuense* distribution as in Ocol and Roncesvalles (Figure 1; Figures 2b, 2c): Salento in the Department of Quindío, and La Línea in the Department of Tolima (Table 2). These two sites are located on the Cordillera Central of Colombia, and have also been disturbed and partially deforested (Table 2). Thus, they served as appropriate scenarios in which we could extend the use of our growth model. Demographic data were not obtained from these two sites. These were included as independent sites for age dating (see sections below). 

**Table 2:** Summary of information retrieved during interviews with locals.

**Site; Coordinates (NW corner of Figure 2); altitudinal range (m)**	**Timing of deforestation**	**Deforestation procedure**	**Economy before**	**Economy today**
Roncesvalles, Tolima, Colombia. East slope, Central Cordillera; 75°41’35.37”W 4°07’06.83”N; 2600-2950.	Started in 1905-1920’s; intensified in early 1950’s, rare and selective today	Everything was cut down, including all palms, then the land was burnt for pasture establishment	Large stakeholders with cattle grazing held by hired workers	Medium-size properties with cattle or/and culture plots and other livestock
Ocol, Molinopampa, Amazonas, Peru. East slope of Andes; 77°37’57.74” W 6°13’59.12”S; 2200-2650	Started in 1970’s, intensified in the 1980’s, small-scale, selective today	Forest was cut down except palms, then site was burnt, and houses/plots installed (periodically burnt). Oldest-tallest palms are commonly felled for construction uses	Unassigned (“no man’s”) land	Small to medium-size properties with mixed production systems, cattle grazing with other livestock (pigs, hens, sheep) and fruit, vegetable and grain chagras for family consumption
La Línea, Tolima, Colombia. East slope, Central Cordillera: 75°35’37.77”W 4°30’47.55”N; 2500-2700	Started in 1905-1920’s, intensified in early 1950’s, rare or absent today	Everything was cut down, including all palms, then the land was burnt for pasture establishment	Large stakeholders with cattle grazing held by hired workers	Same as before, or Medium-size properties with cattle or/and culture plots and other livestock
Salento, Quindío, Colombia. West slope, Central Cordillera; 75°35’57.76”W 4°41’40.28”N; 2400-2700	Started 1840- 1900’s, intensified during first half of 1900’s, rare or absent today	The forest was cut down and the land was burnt for pasture installment, except for some palms that were left standing as a construction material source	Large stakeholders with cattle grazing held by hired workers	Large stakeholders with cattle grazing held by hired workers

#### Ethics statements

No permits were required for the described study, which complied with all relevant regulations. The lands accessed are privately owned; owners were previously contacted and voluntarily agreed on access to their properties and on activities implied in the described study. No live or preserved collections were retrieved from the field. 

### Demographic protocol

In Ocol and Roncesvalles, we established 60 plots of 20 x 20 m, 40 of them in forest and 20 in pastures (22 in Ocol), thus totaling 122 plots and an area of 4.88 hectares. At each plot, we recorded all individuals of *C. quindiuense* (total: 13352 individuals). We located plots in each habitat from random points located within patches of *C. quindiuense*. From each random point, we measured the distance (R) to the nearest stemmed *C. quindiuense*. This individual served as the proximal corner of the corresponding plot (see 22 for detailed description of the method).

At each plot, we measured the following variables: slope gradient (averaged from three measures taken with a Suunto PM5/1520 Clinometer); (2) the coordinates at the center of plot to ensure that the minimal distance between plots was 50 m, thus limiting the effects of autocorrelation in our sampling; (3) the diameter at breast height (DBH, cm) of all trees with DBH > 10 cm, including *C. quindiuense*, in order to test the influence of forest structure on *Ceroxylon*’s demography. The area of the plots was not adjusted for slope, as many of the individuals (i.e. seedlings, juveniles 1, juveniles 2) depend more on the area of the substrate than on the projected horizontal. Also, we wanted to follow Anthelme et al. (22) who did not adjust for slope, in order to allow other authors to make interspecific comparisons. We calculated basal areas of all trees (m^2^/ha), and of *C. quindiuense* in particular, from DBH data, in order to estimate the dominance of *C. quindiuense*. Differences in diameters were assessed using T-tests to compare the mean values for plots.

**Figure 3:**
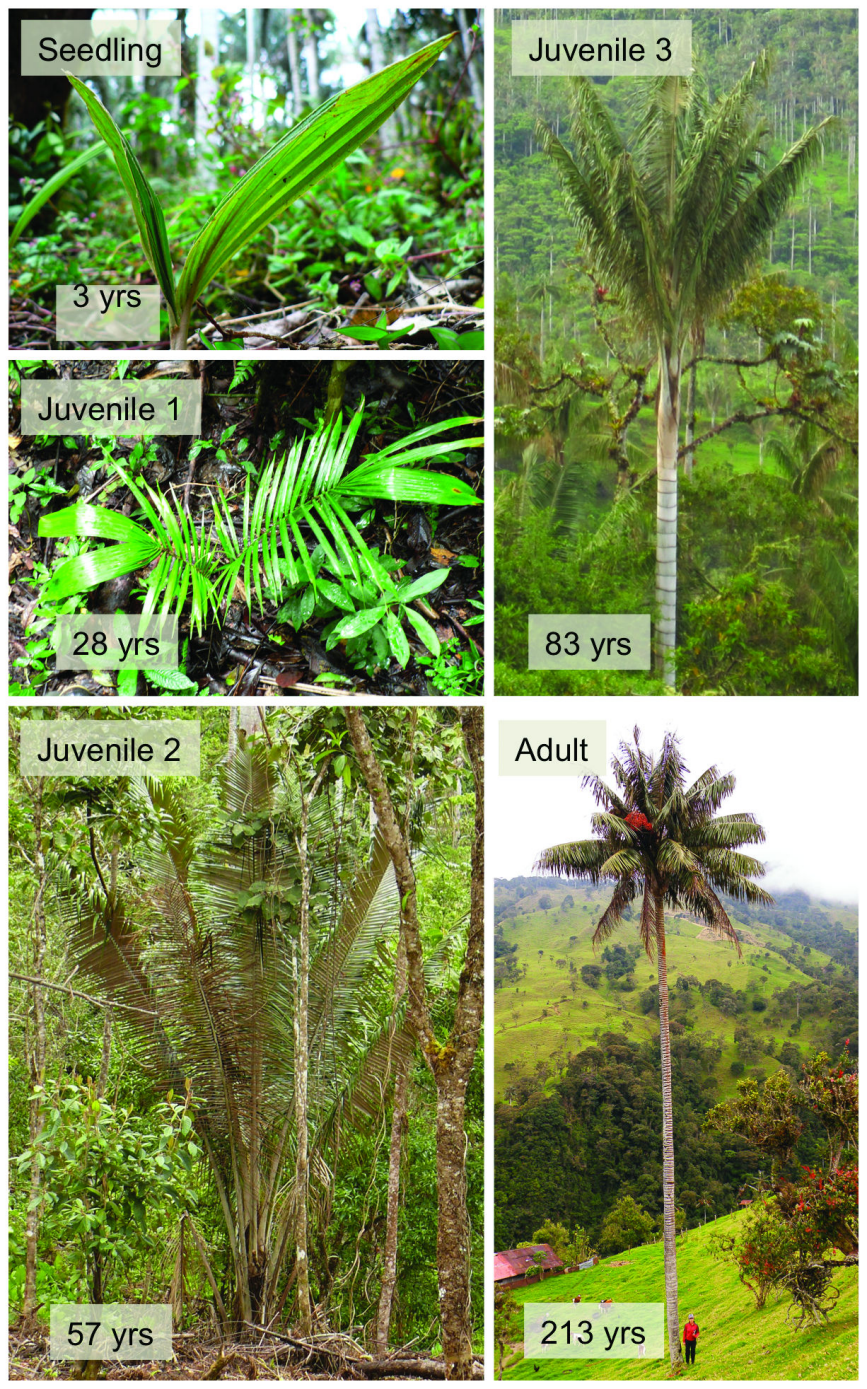
Life stages assigned to *Ceroxylon quindiuense* populations, after [22]: Seedling, Juvenile 1, Juvenile 2 (or “large rosettes”), Juvenile 3, and Adult. The bottom-left number indicates the estimated age by the end of that developmental phase, after [36].

Demographic data on *C. quindiuense* were obtained by counting the total number of individuals. Each individual was assessed as belonging to one of five life stages (Figure 3), following Anthelme et al. [22]:

• Seedling (S): undivided (lanceolate) leaves
• Juvenile 1 (J1): divided leaves (at least 2 pinnae); length < 2 m, no stem
• Juvenile 2 (J2): leaf length > 2 m, no stem. These will be referred to also as “large rosettes”, compared to J1, which are smaller rosettes. This phase ranges from age 28-57 yr in *C. alpinum* [34].
• Juvenile 3 (J3): with stem but no evidence of reproduction, leaf scars parallel, crown erect;
• Adult (A): evidence of reproduction (fertile, or with dead, hanging inflorescences or pieces of them, or diagonal and intersecting leaf scars on the stem); crown hemispherical.

Seedlings have a distinct foliar morphology and represent the germination and early development phase. Following the seedling phase, the rosette phase is long-lasting (57 years in average in *C. alpinum* in forest conditions, [34]) and is better subdivided in two classes (J1 and J2). Rosette development is evidenced by increase in rachis length and pinnae number, but since counting pinnae in the field is unpractical, the length of the leaf served as class limit indicator. J3 are morphologically distinct as they develop aboveground stems but have not yet reproduced. They represent a short transition phase characterized by fast increase in height. Then the adult phase is very long lasting (over 100 years in *C. alpinum*), but could not be successfully sub-divided within the demographic protocol used here because the forest canopy is too closed and leaf scars are too numerous to allow detailed observations on the stem. Adult individuals were assessed as either male or female when reproductive evidence was available. The raw data were entered in Datasets S1 and S2 (Supporting Information). 

### Age dating protocol

In parallel with the demographic study, we intended to infer the relationship between the history of deforestation and the age of the remaining adult palms in pastures. Age measurement of adult palms was designed to examine age variation in adult populations of *C. quindiuense*. A large variation in adult populations would reveal the presence of several generations of palms, thus truly resembling an adult population that established before deforestation and that had been spared form logging. In contrast, a reduced variation in the age of adult populations would increase the probability that it is represented by only one generation, likely coming from rosettes. For this purpose, at the four sites we calculated the age of 30 to 43 adults (total: 154 individuals studied). Age estimations were based on a modified version of Corner’s model [35], which combines number of leaf scars and leaf production rate. In order to count leaf scars and measure height we took high-resolution photographs of adult palms selected by the method of the nearest neighbor, starting at a randomly chosen point. A person stood by the palms as a scale. In order to minimize distortion caused by the spherical aberration of the camera lens, we photographed each palm from a point lying at an elevation corresponding to the middle height of the stem. Stem measurements were made on the photographs with ImageJ64 software [36]. The age of juveniles with no visible aerial stem was estimated based on leaf size using the growth parameters of the morphologically similar *C. alpinum* [34]. This estimate is consistent with the duration of the establishment phase in other long-lived palms, such as *Rhopalostylis sapida*, which takes 43 years to develop an aboveground stem [37], or *Sabal palmetto* that takes between 30 and 60 years to complete this phase [38].

According to our model, the age of an individual is estimated as: 

Age = 57.3 + (N/ 4.89) + (L∗35.78 / 11)

where

N: Number of leaf scars before the first reproduction

L: Distance from the first reproduction to the crown (in meters)

The constant 57.3 is the maximal duration in years of the rosette phase, and the constant 11 is the number of leaves produced per year during the reproductive phase, both parameters as estimated for *C. alpinum* by Vergara-Chaparro [34]. We use the data of Vergara-Chaparro as the only consistent age data available for *Ceroxylon* spp. so far, even if these were done in forests. In pastures, variation in age is expected to be more fluctuating resulting from a series of events that can be are hardly tracked in such longevous organisms (burning, logging, herbivory). Therefore, while acknowledging these contingencies, we assumed that the duration of the rosette phase in pasture would be equivalent to the maximum measurement obtained in forests. This, because we considered growth in pastures as being far from optimum, as in closed forests where light is scarce. The constant 4.89 is the average leaf production rate for juveniles of *C. quindiuense*, as counted in cultivated palms of this species and of the closely related *C. ventricosum* [39]. Finally the constant 35.78 corresponds to the average number of scars per meter in the uppermost portion of the stem. As the scars in the reproductive portion of the stem are too closely packed, and far away from ground level, it is difficult to count the scars on standing palms. We used an empirical constant obtained from direct counts on the reproductive portions of nine stems found dead on the ground in Salento, on which scars could be unambiguously counted. We do not account for variability underlying these constants as we consider it unimportant with respect to both the attainable precision of the models, and the general purpose of age estimation within this specific study. 

Since height at the first reproduction was put into the growth model, and is considered an indicator of exposure to light during growth [40], this figure was compared among sites. 

### Land use history

In order to test our hypothesis that the species has been to some degree resilient to deforestation, and other forms of ecosystem degradation such as fire and herbivory, we needed to gather as much information as possible about the deforestation history and the activities that have been carried out within the sites where the palms stand. As a proxy of the history of land use of each area, we estimated the percentage of forest cover at each site by computing the total area of forested fragments with ImageJ64 software on screenshot images obtained from GoogleEarth (Peruvian site) and the Instituto Geográfico Agustín Codazzi (IGAC) (http://geoportal.igac.gov.co) (Colombian sites), keeping the same scale for each site (1:300000). IGAC images are included in Figure 2(a-c), and the GoogleEarth image was here replaced for an aerial photograph in representation of the Peruvian site. The two sites studied for population ecology displayed contrasting deforestation patterns (Figure 2): whereas deforestation was relatively old and extensive at Roncesvalles (11% of remaining forests), it was much more recent and limited in Ocol (84% of remaining forests). Forest cover was calculated at 46% in Salento, and at 42% in La Línea. Other sources of evidence consisted of the available aerial photographs of Salento (taken in 1955), Roncesvalles (2008), and Ocol (1991), which confirmed in particular that the current state of deforestation in Salento have been achieved before that time. 

During the visits to each site, two to four local inhabitants were interviewed. Experienced adult people were chosen at each site, who had participated in wood extraction-related activities, and who were willing to share their knowledge. Each adult had previously expressed their consent to participate in the interviews. All conditions stated under the categories of research that qualify for exempt status, as stated by the Institutional Review Board, were met. The results of these interviews are summarized in Table 2 as preliminary results. The following were considered relevant issues at each site, and are qualitatively presented: *Timing of deforestation* as remembered by people and in relationship to key events such as the construction of a new road; *Deforestation procedure* if it involves an industrialized process, if it is selective or total, and finally; *Economy before*, and *Economy today*, seeking to portrait the means of subsistence related to land and resource use in the area, both in past and present times. Deforestation first happened in Salento, then in La Línea and Roncesvalles, and finally in Ocol. Deforestation in La Línea and Roncesvalles was total, contracted by large stakeholders who intended to completely clear extensions of land for cattle grazing for the dairy industry. This model also applied for Salento, with the exception that at this site palms were sometimes kept after forest clearing as a construction material to be eventually used. Finally, in Ocol, deforestation was carried out gradually and at a small scale, by subsistence landowners who cleared the forest and left the adult palms standing, saving them as a construction material resource to be gradually used in time. Subsistence at this site was not as biased towards cattle raising as in Colombia, but included other livestock as well as small culture plots for family consumption and/or small, local trade. 

### Statistical analyses

The demographic structure of populations among habitats and sites was assessed with general linear models (GLMs). When taking into account the whole sampling, we used fixed effects models with the variable ‘habitat’ nested within the variable ‘site’ to explain variation in the number of individuals of each life stage in plots. At each site we inferred variations among habitats with two sample T-tests. Analyses were conducted in MINITAB 15 (Minitab Inc. State College, PA) and JMP 7.0 (SAS Institute Inc., Cary, NC) statistical software.

## Results

### Biotic and abiotic characteristics of plots (Table 1)

Palm stems were overall thinner in Ocol than in Roncesvalles (fixed effects model, effect site: p < 0.001). Also, they were significantly thinner in forest than in pasture (fixed effects model, effect habitat within site: p < 0.001). However, when all sites/habitats were compared, no difference was detected between the diameters in the forest of Roncesvalles and those in the pasture of Ocol (two-sample T tests: p > 0.05). We tested for differences in the diameters of the adults and the J3 in the forest of Roncesvalles in order to detect if the increased diameter of the adults of the forests in Roncesvalles (when compared to those of the forests of Ocol) constituted a temporal effect of that specific age class, but these were not significantly different (two-sample T test: p > 0.05). 

Forest plots were located at higher elevation and on steeper terrains than pasture plots (fixed effect models; effect habitat within site: p < 0.001; effect site: p < 0.001), and at the same time they were located at higher elevation (p < 0.001) and on steeper terrain (p < 0.05) in Roncesvalles than in Ocol. The overall basal area of Ocol was higher than that of Roncesvalles (p < 0.001). This was true when comparing forests (1425.92 m^2^/ha ± 488.30 vs. 837.78 m^2^/ha ± 496.28; two-sample T-test: p < 0.001), and when comparing pastures of the two sites (29.63 m^2^/ha ± 28.77 vs. 1.64 m^2^/ha ± 4.05; two-samples T-test: p < 0.001). There was no significant variation in the basal area of *C. quindiuense* among sites (P > 0.05). In the forest, the portion of *C. quindiuense* reached 11.1% of total basal area. In pastures, meanwhile, the portion of *C. quindiuense* was significantly higher in Roncesvalles than in Ocol (90.2% and 40.1%, respectively).

### Demographic structure of palm populations (Roncevalles and Ocol)

**Figure 4:**
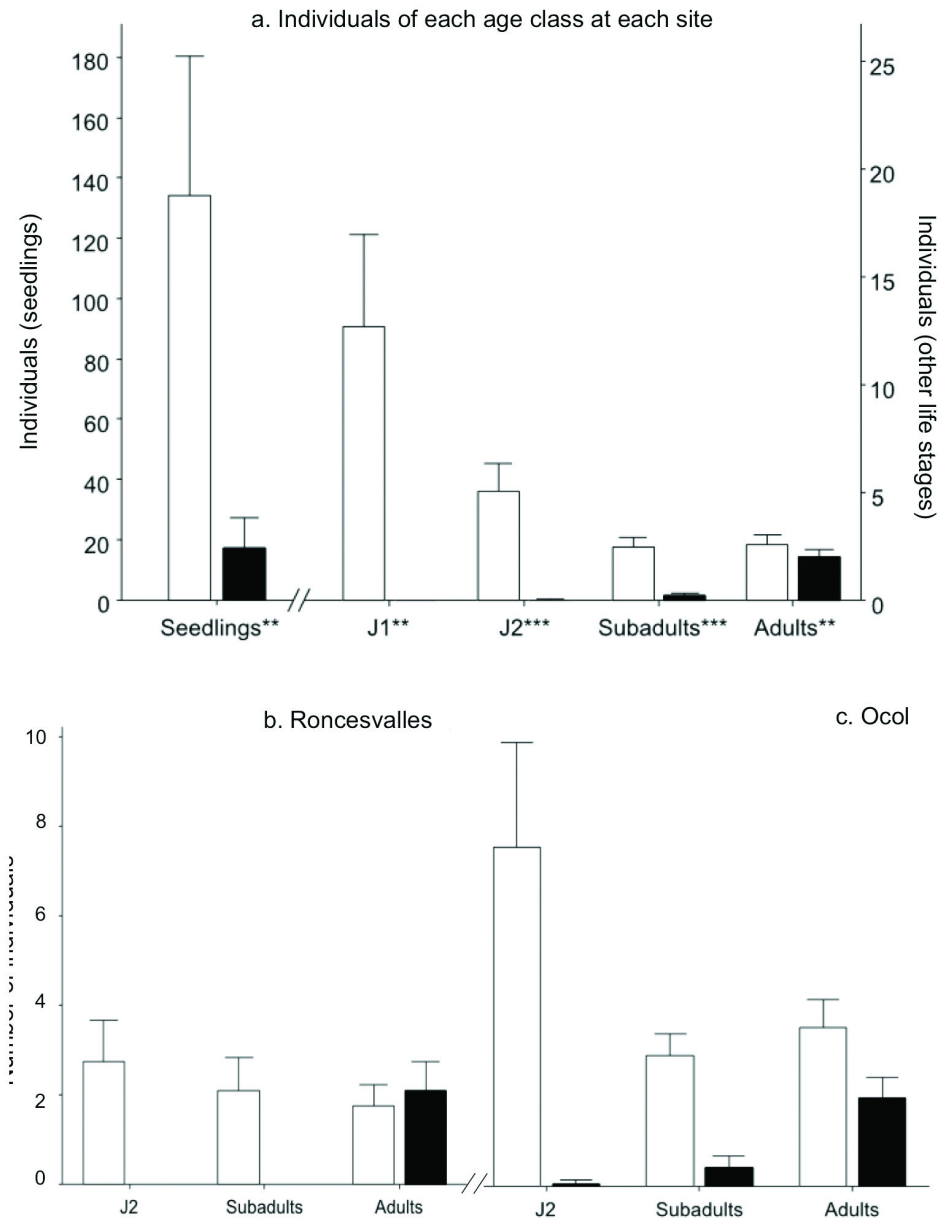
Demographic structure of *Ceroxylon quindiuense* populations in forests (white bars) and pastures (black bars) in Colombia and Peru. (a) Mean number of individuals of each life stage at both sites. Mean number of J2, J3 and adults individuals at Roncesvalles (b) and Ocol (c). Error bars represent 95% CI.

The overall number of individuals of each life stage in pastures and in forests is shown in Figure 4. The overall effects of site variability on palm demography were not significant (fixed effects models: p > 0.05 for each of the life stages). In comparison, the effects of habitat (pasture vs. forest) nested within sites on the number of palm individuals were significant at each life stage, with more individuals found in forest than in pasture for seedlings, J1, adults (fixed effects model: p < 0.01), J2 and J3 (fixed effects model: p < 0.001). In pasture, J1, J2 and J3 (J3) were absent or almost absent (0 ± 0.00, 0.02 ± 0.02 and 0.21 ± 0.08, respectively) whereas they averaged more than two individuals in forest. 

Comparisons between forested plots among sites showed that J2, J3 and adults were overall more abundant in Ocol than in Roncesvalles (4.54 ± 0.51 vs. 2.20 ± 0.25, two-sample T test: p < 0.001). In pastures, each of these life stages was observed in Ocol whereas only adults were observed in Roncesvalles. In Roncesvalles, they were equally distributed between forest and pasture (1.75 ± 0.29 and 2.10 ± 0.37, respectively; two-sample T test: p > 0.05). In comparison, adults were twice as many in the forest as in the pasture in Ocol (3.45 ± 0.36 and 1.91 ± 0.26, respectively; two-sample T test: p < 0.01).

### Age and height at first reproduction of palms in pastures

**Figure 5:**
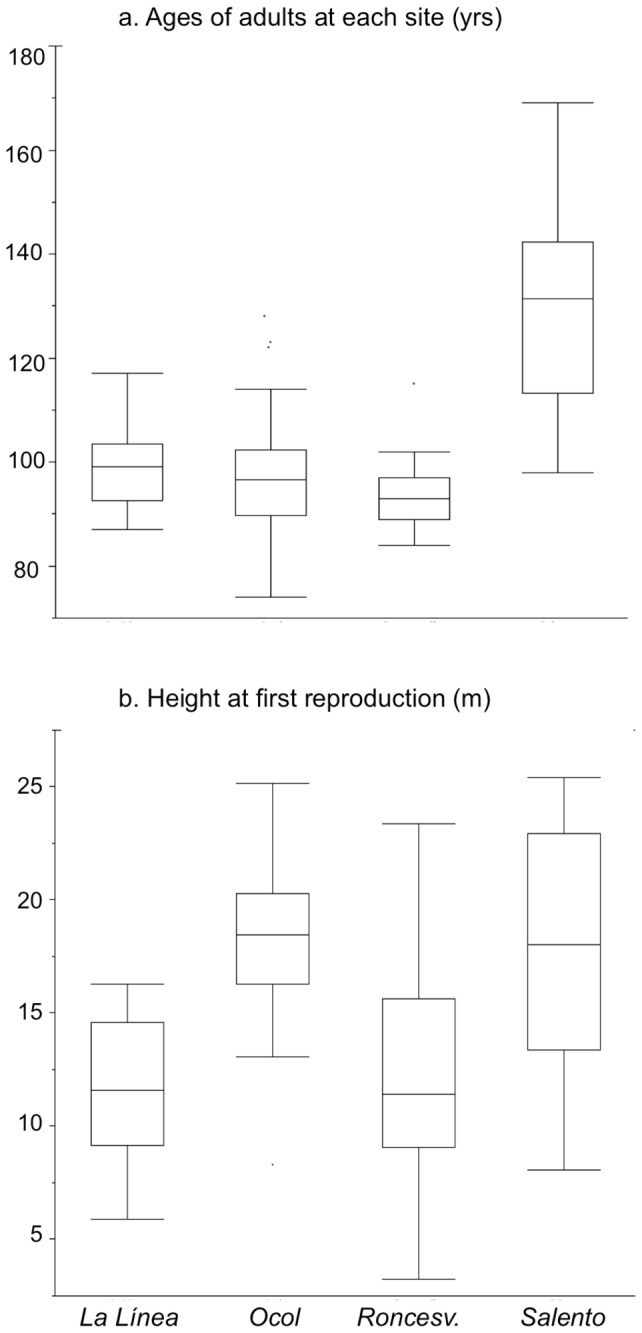
Age distribution of *Ceroxylon quindiuense* adults in Colombia and Peru. (a) age distribution of adults at each site; (b) height at first reproduction at each site (bottom). The box delimits the 25- 75 interquartile range; the central bar indicates the median; outlier symbols = 9^th^ and 91th percentiles. Number of measured individuals are indicated in parentheses: total (154); La Línea (30), Ocol (42), Roncesvalles (43), Salento (39).

Variation in the age (and, therefore, total height) of adults in Salento and Ocol was 2-3 times higher than in La Línea or Roncesvalles (Figure 5a). Adults in La Línea and in Roncesvalles averaged 98 yr ± 7 and 93 yr ± 6, respectively. Adults in Salento were older and averaged 129 yr ± 18. Adults in Ocol were 97 yr ± 13 on average, but several individuals were found to be over 120 years old (Figure 5). Individuals started to reproduce at a greater height in Salento and in Ocol (19.0 m ± 4.9 and 18.6 m ± 3.3, respectively) than in La Línea and Roncesvalles (11.6 m ± 3.0 and 12.2 m ±, 4.6 respectively) (two-sample T tests: p < 0.001) (Figure 5b). 

## Discussion

**Figure 6:**
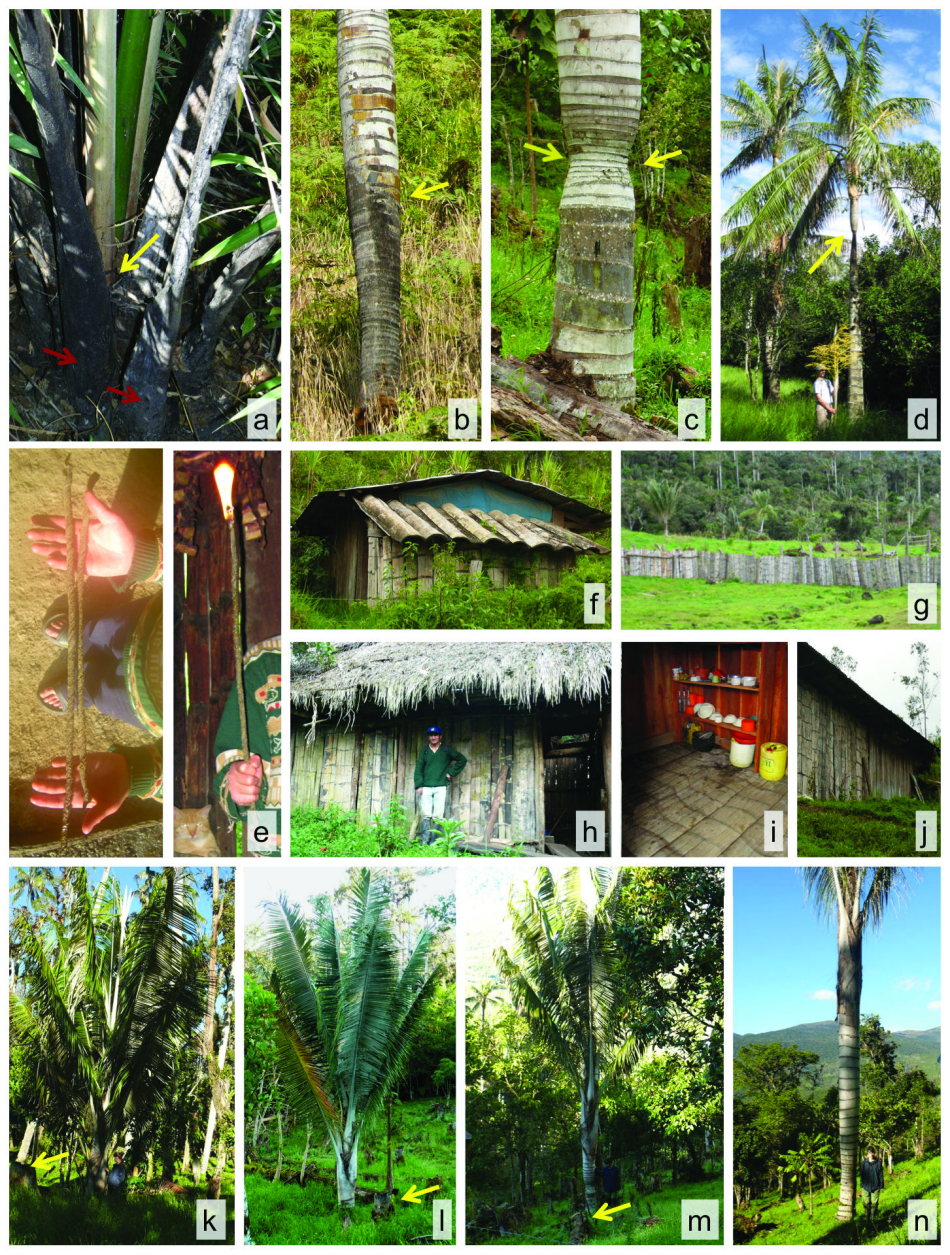
*Ceroxylon quindiuense* in Colombia and Peru, supporting evidence for the proposed scenarios. Evidence of resilience to fire in (a) rosettes (red arrows indicate burnt leaf sheaths, yellow arrow points at green shoot), and (b) stemmed individuals (yellow arrow indicates transition to burnt area of stem); the individual was alive with a full crown. Stem diameter and internode compression in response to fire or mechanical damage in (c) a living J3, and (d) a reproducing adult (arrows point at compressed area). Activities carried out in Ocol that involve the felling of preferably old individuals. (e) Wax extraction for candle production; home construction, including (f) roofs, (h, j) walls, (i) floors, and (g) fences. Sequence of resilient J2 (large rosettes) to J3 growing at a lot of 5 hectares that had been deforested 7.5 years ago and then burnt, as informed by the landowner; (k) vigorous J2 with no stem, (l—n) J3 of different heights (and ages); (k—m) arrows point at pieces of logged palm stems still present at the lot, indicating recent time since deforestation.

As shown previously in another species of *Ceroxylon* (*C. echinulatum*; [22]), the presence of adults in pastures is misleading of the true resilience performed by *C. quindiuense* under deforestation. The U-shaped demographic pattern rather indicates that populations of *C. quindiuense* are virtually dead in this habitat because of absence of regeneration, whereas in the forest all life stages are well represented. Our data, designed to test by which mechanisms residual individuals still occur in pastures, allowed us to understand the respective influences of sparing stemmed individuals and possible rosette resilience (Figure 6a, k-m) on the patterns observed.

### Spared adults and their longevity partially explain patterns of *Ceroxylon* in pastures

**Figure 7:**
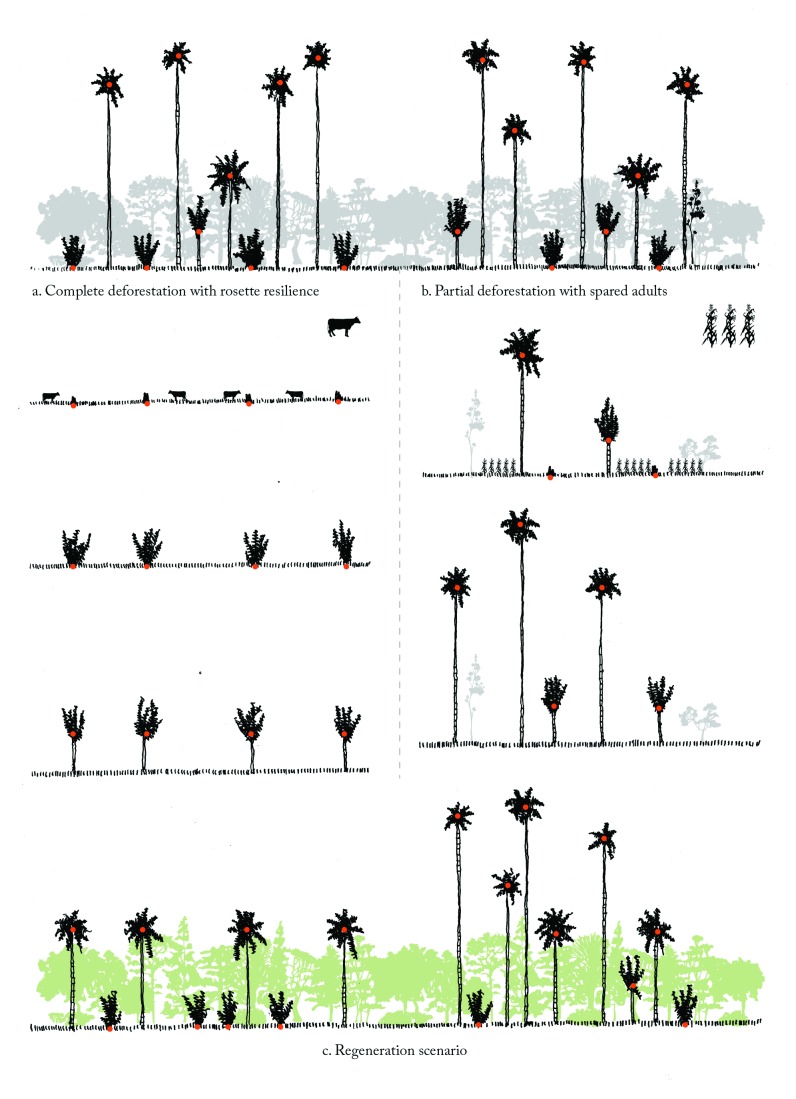
Narrative figure explaining spared adults vs. rosette resilience scenarios. Top: undisturbed forest, with all developmental stages of *Ceroxylon quindiuense*; reading down, left: succession stages following complete deforestation; reading down, right: succession stages following partial deforestation with some spared adults; bottom: explorative prediction of population regeneration, under each of the two deforestation scenarios.

Our data in Ocol and Salento are in line with the hypothesis that, at least, a significant portion of residual adults of *C. quindiuense* still exist in pastures because they have been spared from logging. There, the existence of old palms (up to 169 yr in Salento and up to 123 yr in Ocol) (compare Figure 7a, b), much older than the time when massive deforestation occurred at each site (in the early 1900’s in Salento, in the 1970’ in Ocol), is a proof that not all stemmed palms were cut. At the time of deforestation, these old individuals were adults, juveniles with stems (J3), and some were rosettes. In the specific case of Salento, the extended period since deforestation explains the absence of “young” adults less than 98 years. In Ocol, meanwhile, more recent deforestation explains the presence of relatively young adults. Nevertheless, at both sites, the oldest palms were then gradually extracted for house construction (Table 2; Figure 4e—j). This is the case in Salento at a time when houses were built from *C. quindiuense* stems during the 19^th^ [41,42] and probably during the 20^th^ century, as law did not ban it. In Ocol, the gradual extraction of adult palms in pastures was evidenced (1) by fewer adults within pastures than in forests, (2) by the fact that very old individuals such as those found in Salento were absent, and (3) by the local constructions observed at the site, and the information recovered during interviews.

However, if the “spared adult hypothesis” provides at first sight a consistent explanation for the population structure found in the pastures of Ocol and Salento, it is not sufficient to explain the structure found in the pastures of Roncesvalles and La Línea. Indeed, at these two sites age variation in adults was found particularly narrow, representing only one generation of individuals. This is not consistent with the existence of populations of spared individuals, which, as observed in Ocol and Salento, should hold a larger array of ages (Figure 7b). At Ocol, a fine observation of distribution patterns likely reveals some coherencies with the “spared adult hypothesis”. There, given that the time during which the individual has a resistant subterranean stem is 28—57 years [34], the youngest adults in pastures should be at least 70 years, and only up to 99 years (in order to fit exclusively the “rosette resilience” hypothesis), i.e. 28—57 years + the time since massive deforestation (42 years since 1970). The youngest adult reached 74 years and only 56.8% of them were less than 99 years; thus, the rest were most likely spared. 

### The “rosette resilience” hypothesis adds consistence to the patterns found

Demographic and age data provided in our study support the fact that the “rosette resilience” hypothesis may provide an optimized explanation on adult distribution patterns in pastures, in combination with the “spared adult” hypothesis. Following the time estimates of Vergara-Chaparro [34], the time to produce a large rosette of *Ceroxylon* with underground meristems is at least 28 years. This time combined with time since initial deforestation in Ocol would reach 70 years, which makes possible the presence of adults of 74 years. As well, this would better fit the age of youngest adults in Salento (98 years) than taking into account the “spared adult” hypothesis only. 

In Roncesvalles and La Línea, as supported by interviews conducted at these sites, the most probable scenario was that all adults were cut during massive deforestation (i.e. as opposed to selective, at these sites nothing was spared from logging). This assertion is supported by our data since both sites (1) displayed similar age patterns for adults of *C. quindiuense*, with short age range and absence of adults older than 117 years, and (2) basal area of other trees in Roncesvalles’ pastures was insignificant in comparison with Ocol’s pastures. Consequently, at these two sites the “spared adult” hypothesis is rejected. Meanwhile, the absence of individuals younger than 84 years is in line with the time when massive deforestation took place at these sites, if we take into account resilience of *Ceroxylon* rosettes after deforestation. Subterranean stems start to develop at approximately 28 years (equivalent to end of J1 phase [34]:); taking into account the age of current adult individuals (89-103 years old in both sites) (Figure 5a), these individuals started producing subterranean stems 61—75 years ago. The period is from 1937—1951, which is the time when massive deforestation took place in Roncesvalles and La Línea, thus supporting the hypothesis that rosette resilience is the main mechanism used by *C. quindiuense* to maintain adult populations in pastures at these sites. Only the rosettes, having underground meristems, and juvenile individuals with short stems could survive deforestation, burning and the effects of large herbivores, whereas seedlings and juveniles without underground meristems died (see also 22), and adults were felled. Interestingly, the number of current adults in pastures at Roncesvalles is comparable to the amount of rosettes found in the adjacent forest (Figure 4b). This means that the current pastures harbor the adults that were rosettes with underground meristems at the time of massive deforestation, which reinforces the above hypothesis (Figure 7a).

One might ask, if rosettes are resilient to deforestation, fire, and herbivory, why are they not present in pastures in the Colombian sites, and rarely present in Ocol? The time since deforestation, accounts for this absence. In all cases studied, deforestation began at least 45 years ago, and therefore the vast majority of the persisting rosettes have by now become adults. Furthermore, no regeneration from seedlings has taken place, as this class is not resilient to grazed environments, and therefore no recruitment to J2 and J3 has taken place during this time. However, in rare, recently deforested and periodically burnt lots, rosettes were observed to grow back (Figure 6k—n), and to develop under an open, grazed, environment J3.

Two supplementary data, the height of individuals at first reproduction and the diameter of individuals, sustain our combined hypothesis that both spared adults and rosettes with underground meristems drive the current pattern of adult population of *C. quindiuense* in pastures on a regional scale. In Salento and Ocol, where adults were — at least partially — spared, the height at the first reproduction occurred at 19 m (Figure 5b) at a time when they still grew under forest cover (Table 2). The forest canopy being estimated at 15-30 m in mountain cloud forests [29], this means that *C. quindiuense* either needs to stand higher than the canopy and reach full sunlight in order for reproduction to start. However, at La Línea and Roncesvalles, where all adults were logged during deforestation, first reproduction in pastures occurred at 12 m. The most obvious explanation for this difference is that these palms grew under direct sunlight, accelerating their reproducing cycle under the effects of increased light exposure, as shown for *Attalea speciosa* [40], a palm with a similar developmental model. This supports the “rosette resilience” hypothesis, as these individuals would necessarily come from resilient rosettes. 

Variation in diameter among habitats also supports the “rosette resilience” hypothesis. In most palms, stem diameter is essentially controlled and fixed by apical meristem activity during growth in height, which means that the initial diameter of palm stems remains constant during the life of an individual, unless sustained primary growth occurs [43,44]. We detected no difference between the diameters of J3 and Adults of forests at Roncesvalles, indicating that sustained primary growth does not occur in *C. quindiuense*. Accordingly, the significantly thicker stems of *C. quindiuense* growing in pastures than in forests are additional evidence that they developed their aerial stems after deforestation, taking advantage of direct light exposure. These thicker individuals come from resilient rosettes and not from seedlings or small juveniles, which do not survive deforested environments. The density of adults found in the pastures of Roncesvalles equals the density of J3 in the forests of that site. This adult density resulted of the density of J3 that was present at the time of deforestation. Thus, the surviving J3 of the time are now the adults at the site. Also, this density of adults on pastures is lower that of forests, because the habitat does not allow very young age classes to develop into adults and add to adult density; these adults correspond to a former batch of J3. 

Both the “spared adults” and the “rosette resilience” hypotheses are required to consistently explain the current patterns of *C. quindiuense*’s distribution in pastures, and each can represent more or less relevance at each locality. Our interpretation was possible because we used a multi-site protocol on a regional scale, thus pinpointing the need for studies covering the whole range of target species when analyzing distributional and ecological patterns. Interestingly, a number of other palms have been shown to develop the same pattern in front of massive deforestation, and all of them had a rosette phase with an underground meristem, either similar to that of *C. quindiuense* [22], or developing a “saxophone growth”, which, to some extent, makes them resilient to deforestation, burning and grazing [40,45,46,47]. In contrast, other palms in the tropical Andean mountain forests that do not pass through a rosette establishment phase, including*, Aiphanes erinacea*, *Chamaedorea linearis*, *C. pinnatifrons, Euterpe precatoria*, *Geonoma undata*, *G. orbignyana*, *Prestoea acuminata*, and *Wettinia kalbreyeri*, are seldom observed in deforested areas [48,49]. Because all the above-mentioned examples of palm resilience to deforestation have same mechanism of developing an underground meristem, we recommend this particular mechanism to be integrated in the conceptual framework of ecological resilience of forest species under the multiple effects of deforestation.

### Predictions on the long-term conservation of current populations in pastures

The persistence of adults of *C. quindiuense* in pastures could not by itself prevent the species from local extinction under massive deforestation in all situations. When deforestation is total, it is the high resistance of the rosette life stage, on account of their protected underground meristem, which allows the population to re-establish afterwards, although with an altered population structure (Figure 7c, left side). Therefore, two scenarios of deforestation contrast with a “control” scenario of forest preservation (Figure 7, top strip): complete deforestation (Figure 7a) and deforestation with some adults spared (Figure 7b).

In a sense, the great longevity of *C. quindiuense* might be a drawback because palms surviving in pastures are sometimes so abundant, that they may be taken for well-conserved populations. Also, generation gaps among adults are cryptic, as an age difference of 40-50 years is represented by only a few meters of stem length, scarcely detectable in a palm over 30 m tall. On the other hand, the stature of stemmed palms is often misleading of their age [50]. Accordingly, transmitting a simplified version of the protocol of age dating to local stakeholders would be useful to estimate the current conservation status of populations, and to relate land use history of target areas. If properly managed, the relatively good resilience of *C. quindiuense* to deforestation, combined with its great longevity (at least 169 years, probably more, on account of the maximum age of 213 yr calculated for *C. alpinum* [34]) make up an outstanding asset, which allows populations in pastures to store adult individuals for a long time. The long-term preservation of palms in pastures secures the availability of a large amount of seeds, which may be a crucial driver of population recovery if reforestation ever occurs in a particular area.

What predictions of long-term conservation can we raise for current adult populations surviving in pastures? A crucial requirement for their preservation is that a reforestation event occurs within the life span of residual adults. If so, the shade produced by re-colonizing species should reduce sufficiently water stress and allow seedlings and young juveniles to survive, as shown for *C. echinulatum* [22]. Nevertheless, even under the hypothesis of a reforestation event, the population’s genetic diversity in degraded habitats would be strongly reduced in comparison to natural undisturbed populations, because they would come from a restricted number of female individuals, probably resulting from a few reproductive events [51]. Under the scenario where some adults were spared from logging, the more numerous individuals and the fact that they represent a wider age-range would lead to a greater genetic diversity than under the scenario where all initial adults were cut (Figure 7b, as opposed to 7a), as they represent several reproductive events. Furthermore, another means by which overall genetic diversity could be affected and which remains to be studied is the effect of a precocious start of reproduction triggered by exposure to light in the juvenile phases. Our data unambiguously suggest that palms growing in pastures start reproducing at a lower height than those growing in forests. The effect of an accelerated life history on demography could be negative if a higher allocation to fecundity decreases adult survival or longevity [52], but positive if it allows earlier seedling recruitment that serves as a population rescue mechanism following a deforestation event. However these hypotheses are speculative so far, and how these demographic fluctuations between sites affect the distribution of genetic variation within and among populations is a topical scientific challenge, which, to our knowledge, has been studied in only a few cases for Andean plants [53,54] and only once with a conservation perspective [55]. 

The resilience mechanisms discussed here might explain the maintenance and high population density of other palm species, such as *Attalea butyracea*, *Acrocomia aculeata*, and *Oenocarpus bataua*, in intervened tropical ecosystems. Like *Ceroxylon*, populations of these species often occupy subsets of the optimal available habitat, forming dense stands not easily explained by solely climatic or edaphic variables. Their patchy distribution could possibly be attributed to differential resilience mechanisms with regard to other sympatric species, an issue that should be further addressed. 

As a final remark, we evoke uniformitarianism principles that state: “the present is the key to the past” [56]; mechanisms that allow *Ceroxylon* to persist today amidst vast anthropogenic disturbances in the cloud forests of the Andes may have been also responsible for their survival and recolonization in the past. The long-term genetic and ecological effects of the associated demographic fluctuations deserve further research.

## Supporting Information

Dataset S1 **Demography of *Ceroxylon quindiuense* in plots at Roncevalles (Colombia) and Ocol (Peru).**
(XLSX)

Dataset S2 **Aggregation of *Ceroxylon quindiuense* in plots at Roncevalles (Colombia) and Ocol (Peru).**
(XLS)
